# Crystal structure of pyrazoxyfen

**DOI:** 10.1107/S2056989015023233

**Published:** 2015-12-09

**Authors:** Eunjin Kwon, Jineun Kim, Gihaeng Kang, Tae Ho Kim

**Affiliations:** a Department of Chemistry and Research Institute of Natural Sciences, Gyeongsang National University, Jinju 52828, Republic of Korea

**Keywords:** crystal structure, pyrazoxyfen, aceto­phenone, herbicide

## Abstract

The title compound, C_20_H_16_Cl_2_N_2_O_3_ (systematic name: 2-{[4-(2,4-di­chloro­benzo­yl)-1,3-di­methyl­pyrazol-5-yl}­oxy}-1-phenyl­ethan-1-one), is the benzoyl­pyrazole herbicide pyrazoxyfen. The asymmetric unit comprises two independent mol­ecules, *A* and *B*, in which the pyrazole ring makes dihedral angles of 80.29 (10) and 61.70 (10)° and 87.60 (10) and 63.92 (8)°, respectively, with the di­chloro­phenyl and phenyl rings. In the crystal, C—H⋯O and C—H⋯N hydrogen bonds, and C—H⋯π and π–π [3.646 (2) Å] inter­actions link adjacent mol­ecules, forming a two-dimensional network parellel to (011). In addition, the networks are linked by weak inter­molecular C—Cl⋯π [3.356 (2), 3.950 (2), 3.250 (2) and 3.575 (2) Å] inter­actions, resulting in a three-dimensional architecture.

## Related literature   

For information on the herbicidal properties of the title compound, see: Hirai *et al.* (2002 ▸). For a related crystal structure, see: Indumathi *et al.* (2012 ▸).
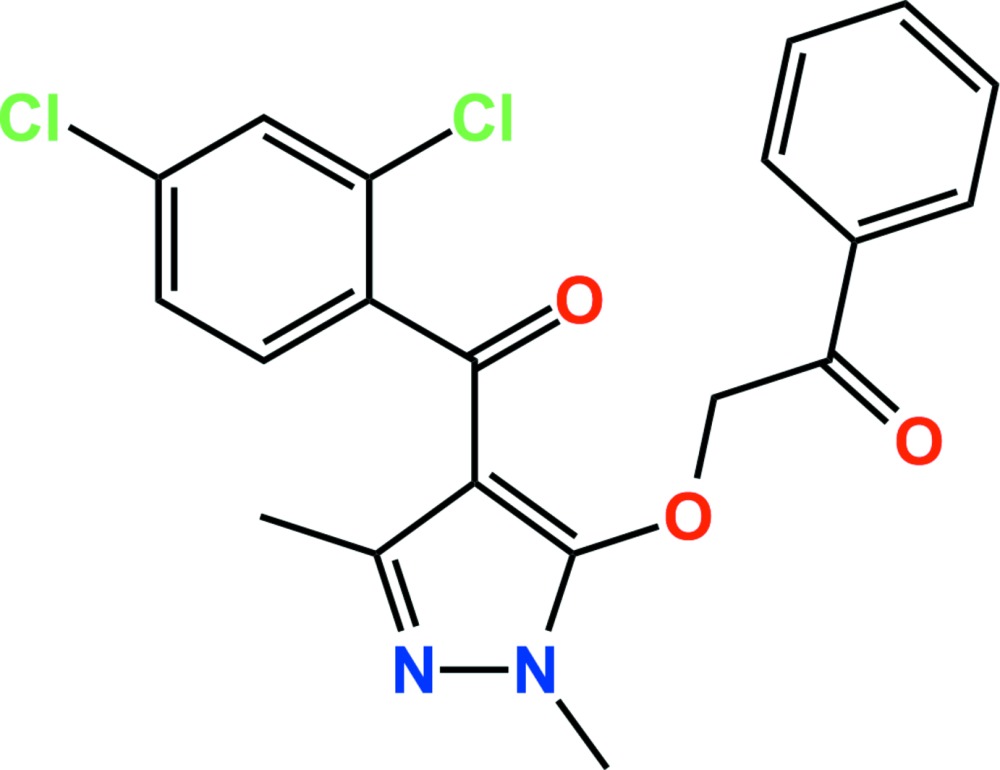


## Experimental   

### Crystal data  

C_20_H_16_Cl_2_N_2_O_3_
*M* *_r_* = 403.25
Triclinic, 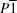

*a* = 7.827 (3) Å
*b* = 15.534 (5) Å
*c* = 15.886 (6) Å
α = 88.82 (2)°
β = 89.093 (18)°
γ = 77.266 (18)°
*V* = 1883.4 (11) Å^3^
*Z* = 4
Mo *K*α radiation
μ = 0.37 mm^−1^
*T* = 173 K
0.20 × 0.16 × 0.11 mm

### Data collection  

Bruker APEXII CCD diffractometer
Absorption correction: multi-scan (*SADABS*; Bruker, 2014 ▸) *T*_min_ = 0.684, *T*_max_ = 0.746
22903 measured reflections
6579 independent reflections
3991 reflections with *I* > 2σ(*I*)
*R*_int_ = 0.063

### Refinement  

*R*[*F* ^2^ > 2σ(*F* ^2^)] = 0.049
*wR*(*F* ^2^) = 0.109
*S* = 0.99
6579 reflections
491 parameters
H-atom parameters constrained
Δρ_max_ = 0.25 e Å^−3^
Δρ_min_ = −0.35 e Å^−3^

### 

Data collection: *APEX2* (Bruker, 2014 ▸); cell refinement: *SAINT* (Bruker, 2014 ▸); data reduction: *SAINT*; program(s) used to solve structure: *SHELXS97* (Sheldrick, 2008 ▸); program(s) used to refine structure: *SHELXL2013* (Sheldrick, 2015 ▸); molecular graphics: *DIAMOND* (Brandenburg, 2010 ▸); software used to prepare material for publication: *SHELXTL* (Sheldrick, 2008 ▸).

## Supplementary Material

Crystal structure: contains datablock(s) global, I. DOI: 10.1107/S2056989015023233/hg5466sup1.cif

Structure factors: contains datablock(s) I. DOI: 10.1107/S2056989015023233/hg5466Isup2.hkl

Supporting information file. DOI: 10.1107/S2056989015023233/hg5466Isup3.cml

**Fig. 1.**
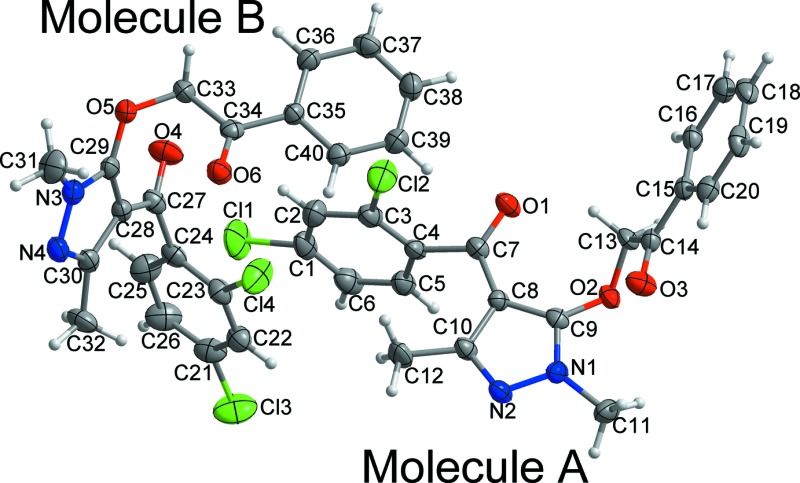
The asymmetric unit of the title compound with the atom-numbering scheme. Displacement ellipsoids are drawn at the 50% probability level. H atoms are shown as small spheres of arbitrary radius.

**Fig. 2.**
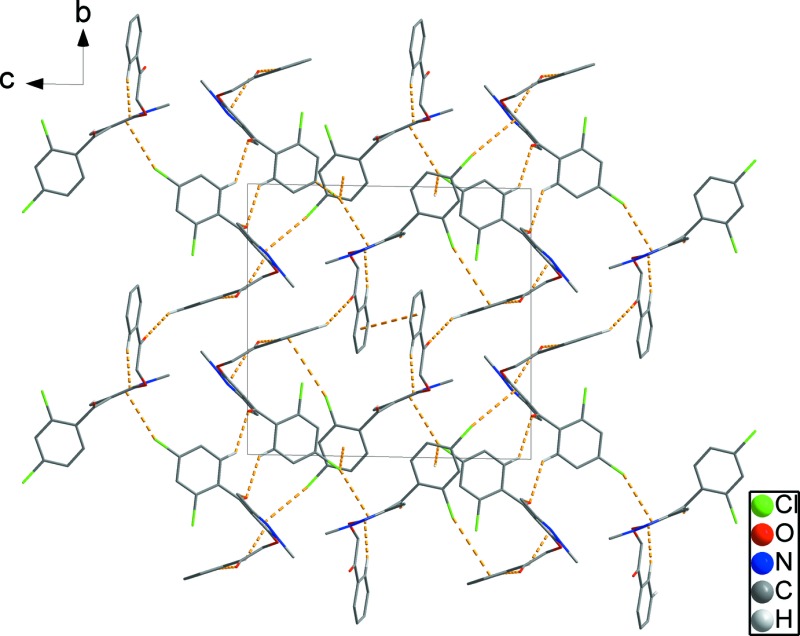
Crystal packing viewed along the a axis. The intermolecular interactions are shown as dashed lines.

. DOI: 10.1107/S2056989015023233/hg5466fig1.tif
The asymmetric unit of the title compound with the atom-numbering scheme. Displacement ellipsoids are drawn at the 50% probability level. H atoms are shown as small spheres of arbitrary radius.

a . DOI: 10.1107/S2056989015023233/hg5466fig2.tif
Crystal packing viewed along the *a* axis. The inter­molecular inter­actions are shown as dashed lines.

CCDC reference: 1440271

Additional supporting information:  crystallographic information; 3D view; checkCIF report

## Figures and Tables

**Table 1:** Hydrogen-bond geometry (Å, °) *Cg*3 and *Cg*6 are the centroids of the C15–C20 and C35–C40 rings, respectively.

*D*—H⋯*A*	*D*—H	H⋯*A*	*D*⋯*A*	*D*—H⋯*A*
C12—H12*A*⋯O1^i^	0.98	2.42	3.353 (4)	159
C32—H32*A*⋯O4^i^	0.98	2.45	3.420 (4)	168
C16—H16⋯N2^ii^	0.95	2.51	3.414 (4)	159
C36—H36⋯N4^ii^	0.95	2.54	3.334 (4)	141
C37—H37⋯O6^ii^	0.95	2.55	3.490 (4)	172
C25—H25⋯O4^iii^	0.95	2.56	3.302 (4)	135
C39—H39⋯O3^iv^	0.95	2.54	3.346 (4)	143
C33—H33*A*⋯*Cg*6^v^	0.99	2.97	3.683 (3)	130
C38—H38⋯*Cg*3^vi^	0.95	2.68	3.499 (3)	145

Symmetry codes: (i) 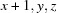; (ii) 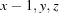; (iii) 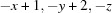; (iv) 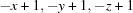; (v) 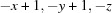; (vi) 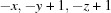.

## supplementary crystallographic information

### S1. Comment

Pyrazoxyfen [systematic name: 2-[4-(2,4-dichlorobenzoyl)-1,3-dimethylpyrazol-5-yloxy]acetophenone] is a the benzoyl pyrazole herbicides. Various pyrazole derivatives with potent herbicidal activity have been synthesized and some are in use as herbicides such as pyrazolate, pyrazoxyfen, benzofenap, pyraflufen-ethyl, fluazolate and pyrazosulfuron-ethyl (Hirai *et al.*, 2002). The asymmetric unit comprises two independent molecules, A and B, in which the dihedral angle between the dichlorophenyl and pyrazole and phenyl ring planes are 80.29 (10), 61.70 (10), 87.60 (10), and 63.92 (8)°, respectively. All bond lengths and bond angles are normal and comparable to those observed in similar crystal structures (Indumathi *et al.*, 2012).

In the crystal structure (Fig. 2), C–H···O and C–H···N hydrogen bonds, C–H···π (Table 1), and *Cg*3···*Cg*3^vi^, 3.646 (2) Å (*Cg*3 is the centroid of the C15—C20 ring) interactions link adjacent molecules, forming a two-dimensional network parelle to (011) plane. In addition, the networks are linked by weak intermolecular C1–Cl1···*Cg*4^iii^, 3.356 (2), C3–Cl2···*Cg*6, 3.950 (2), C21–Cl3···*Cg*1^vii^, 3.250 (2) and C21–Cl3···*Cg2*^i^, 3.575 (2) Å (*Cg*1, *Cg2, *Cg**4 and *Cg*6 are the centroids of the N1—N2—C8—C9—C10, C1—C6, N3—N4—C28—C29—C30 and C35–C40 rings) interactions, resulting in a three-dimensional architecture [for Symmetry codes (i) *x* + 1, *y*, *z*; (ii) *x* − 1, *y*, *z*; (iii) −*x* + 1, −*y* + 2, −*z*; (iv) −*x* + 1, −*y* + 1, −*z* + 1; (v) −*x* + 1, −*y* + 1, −*z*; (vi) −*x*, −*y* + 1, −*z* + 1, (vii) −*x* + 1, −*y* + 2, −*z* + 1].

### S2. Experimental

The title compound was purchased from the Dr. Ehrenstorfer GmbH Company. Slow evaporation of a solution in CH_3_CN gave single crystals suitable for X-ray analysis.

### S3. Refinement

All H-atoms were positioned geometrically and refined using a riding model with d(C—H) = 0.95 Å, *U*_iso_ = 1.2*U*_eq_(C) for aromatic C—H, d(C—H) = 0.99 Å, *U*_iso_ = 1.2*U*_eq_(C) for methylene C—H and d(C—H) = 0.98 Å, *U*_iso_ = 1.5*U*_eq_(C) for methyl C—H.

### Crystal data

**Table:**

C_20_H_16_Cl_2_N_2_O_3_	*Z* = 4
*M**_r_* = 403.25	*F*(000) = 832
Triclinic, *P*1	*D*_x_ = 1.422 Mg m^−^^3^
*a* = 7.827 (3) Å	Mo *K*α radiation, λ = 0.71073 Å
*b* = 15.534 (5) Å	Cell parameters from 3560 reflections
*c* = 15.886 (6) Å	θ = 2.6–21.7°
α = 88.82 (2)°	µ = 0.37 mm^−^^1^
β = 89.093 (18)°	*T* = 173 K
γ = 77.266 (18)°	Plate, colourless
*V* = 1883.4 (11) Å^3^	0.20 × 0.16 × 0.11 mm

### Data collection

**Table:**

Bruker APEXII CCD diffractometer	3991 reflections with *I* > 2σ(*I*)
φ and ω scans	*R*_int_ = 0.063
Absorption correction: multi-scan (*SADABS*; Bruker, 2014)	θ_max_ = 25.0°, θ_min_ = 1.3°
*T*_min_ = 0.684, *T*_max_ = 0.746	*h* = −7→9
22903 measured reflections	*k* = −18→18
6579 independent reflections	*l* = −18→18

### Refinement

**Table:**

Refinement on *F*^2^	0 restraints
Least-squares matrix: full	Hydrogen site location: inferred from neighbouring sites
*R*[*F*^2^ > 2σ(*F*^2^)] = 0.049	H-atom parameters constrained
*wR*(*F*^2^) = 0.109	*w* = 1/[σ^2^(*F*_o_^2^) + (0.0415*P*)^2^] where *P* = (*F*_o_^2^ + 2*F*_c_^2^)/3
*S* = 0.99	(Δ/σ)_max_ = 0.001
6579 reflections	Δρ_max_ = 0.25 e Å^−^^3^
491 parameters	Δρ_min_ = −0.35 e Å^−^^3^

### Special details

**Table:**

Geometry. All e.s.d.'s (except the e.s.d. in the dihedral angle between two l.s. planes) are estimated using the full covariance matrix. The cell e.s.d.'s are taken into account individually in the estimation of e.s.d.'s in distances, angles and torsion angles; correlations between e.s.d.'s in cell parameters are only used when they are defined by crystal symmetry. An approximate (isotropic) treatment of cell e.s.d.'s is used for estimating e.s.d.'s involving l.s. planes.

### Fractional atomic coordinates and isotropic or equivalent isotropic displacement parameters (Å^2^)

**Table:**

	*x*	*y*	*z*	*U*_iso_*/*U*_eq_	
Cl1	0.24743 (13)	1.11847 (6)	0.20923 (6)	0.0721 (3)	
Cl2	0.24441 (12)	0.77988 (5)	0.27588 (5)	0.0560 (3)	
Cl3	0.75954 (12)	1.04970 (5)	0.32607 (6)	0.0662 (3)	
Cl4	0.79186 (13)	0.73468 (5)	0.19305 (5)	0.0597 (3)	
O1	0.0216 (3)	0.81955 (12)	0.45887 (12)	0.0409 (5)	
O2	0.1419 (2)	0.74996 (11)	0.64187 (10)	0.0298 (5)	
O3	0.2716 (3)	0.57587 (12)	0.63891 (12)	0.0410 (5)	
O4	0.5267 (3)	0.83966 (12)	0.00376 (13)	0.0456 (5)	
O5	0.7107 (2)	0.67719 (12)	−0.10634 (11)	0.0352 (5)	
O6	0.7996 (3)	0.58373 (11)	0.03822 (11)	0.0357 (5)	
N1	0.4384 (3)	0.74396 (13)	0.63553 (13)	0.0295 (6)	
N2	0.5663 (3)	0.76683 (14)	0.58592 (14)	0.0324 (6)	
N3	0.9906 (3)	0.69469 (14)	−0.10444 (13)	0.0326 (6)	
N4	1.1021 (3)	0.74130 (15)	−0.06989 (14)	0.0345 (6)	
C1	0.2381 (4)	1.03478 (19)	0.2818 (2)	0.0414 (8)	
C2	0.2477 (4)	0.9515 (2)	0.25301 (19)	0.0441 (8)	
H2	0.2663	0.9389	0.1949	0.053*	
C3	0.2296 (4)	0.88565 (17)	0.31104 (18)	0.0357 (7)	
C4	0.2039 (3)	0.90297 (17)	0.39544 (17)	0.0283 (7)	
C5	0.2022 (4)	0.98763 (17)	0.42230 (18)	0.0359 (7)	
H5	0.1901	0.9999	0.4807	0.043*	
C6	0.2175 (4)	1.05413 (19)	0.3659 (2)	0.0425 (8)	
H6	0.2140	1.1121	0.3847	0.051*	
C7	0.1674 (4)	0.83569 (16)	0.45857 (16)	0.0283 (7)	
C8	0.3040 (3)	0.79810 (15)	0.51804 (16)	0.0245 (6)	
C9	0.2832 (4)	0.76044 (16)	0.59716 (17)	0.0264 (6)	
C10	0.4856 (4)	0.79928 (17)	0.51579 (17)	0.0296 (7)	
C11	0.4789 (4)	0.71264 (19)	0.72142 (17)	0.0423 (8)	
H11A	0.3756	0.7322	0.7576	0.063*	
H11B	0.5760	0.7366	0.7420	0.063*	
H11C	0.5125	0.6480	0.7225	0.063*	
C12	0.5910 (4)	0.8278 (2)	0.44605 (18)	0.0424 (8)	
H12A	0.7152	0.8132	0.4610	0.064*	
H12B	0.5528	0.8917	0.4368	0.064*	
H12C	0.5744	0.7972	0.3945	0.064*	
C13	0.0333 (3)	0.69531 (16)	0.60951 (16)	0.0284 (7)	
H13A	0.0133	0.7088	0.5488	0.034*	
H13B	−0.0818	0.7087	0.6388	0.034*	
C14	0.1167 (4)	0.59876 (17)	0.62135 (16)	0.0270 (7)	
C15	0.0051 (4)	0.53405 (17)	0.60969 (15)	0.0267 (7)	
C16	−0.1689 (4)	0.56036 (18)	0.58556 (16)	0.0317 (7)	
H16	−0.2190	0.6213	0.5764	0.038*	
C17	−0.2700 (4)	0.49842 (19)	0.57471 (16)	0.0368 (7)	
H17	−0.3888	0.5168	0.5581	0.044*	
C18	−0.1972 (4)	0.4100 (2)	0.58811 (17)	0.0408 (8)	
H18	−0.2654	0.3674	0.5801	0.049*	
C19	−0.0247 (4)	0.38339 (18)	0.61323 (17)	0.0390 (8)	
H19	0.0242	0.3225	0.6234	0.047*	
C20	0.0767 (4)	0.44465 (17)	0.62359 (16)	0.0343 (7)	
H20	0.1954	0.4258	0.6402	0.041*	
C21	0.7426 (4)	0.98693 (19)	0.23920 (19)	0.0403 (8)	
C22	0.7747 (4)	0.89714 (18)	0.24907 (18)	0.0365 (7)	
H22	0.8092	0.8695	0.3019	0.044*	
C23	0.7556 (4)	0.84838 (17)	0.18030 (18)	0.0337 (7)	
C24	0.7060 (3)	0.88679 (17)	0.10255 (18)	0.0311 (7)	
C25	0.6756 (4)	0.97795 (18)	0.0948 (2)	0.0437 (8)	
H25	0.6406	1.0059	0.0422	0.052*	
C26	0.6957 (4)	1.02854 (19)	0.1629 (2)	0.0478 (9)	
H26	0.6775	1.0909	0.1573	0.057*	
C27	0.6781 (4)	0.83393 (17)	0.02736 (17)	0.0299 (7)	
C28	0.8278 (3)	0.78297 (16)	−0.01511 (16)	0.0259 (6)	
C29	0.8295 (4)	0.71799 (17)	−0.07395 (16)	0.0281 (7)	
C30	1.0045 (4)	0.79338 (18)	−0.01446 (17)	0.0309 (7)	
C31	1.0567 (4)	0.6240 (2)	−0.16135 (18)	0.0511 (9)	
H31A	1.0866	0.5680	−0.1296	0.077*	
H31B	1.1616	0.6348	−0.1904	0.077*	
H31C	0.9669	0.6209	−0.2028	0.077*	
C32	1.0887 (4)	0.85251 (19)	0.03547 (19)	0.0454 (8)	
H32A	1.2127	0.8436	0.0193	0.068*	
H32B	1.0789	0.8387	0.0956	0.068*	
H32C	1.0300	0.9141	0.0244	0.068*	
C33	0.5743 (4)	0.65695 (17)	−0.05442 (16)	0.0324 (7)	
H33A	0.5078	0.6219	−0.0870	0.039*	
H33B	0.4926	0.7126	−0.0385	0.039*	
C34	0.6429 (4)	0.60573 (16)	0.02483 (16)	0.0278 (7)	
C35	0.5102 (3)	0.58522 (16)	0.08449 (16)	0.0259 (6)	
C36	0.3373 (4)	0.59303 (17)	0.06168 (17)	0.0326 (7)	
H36	0.3012	0.6133	0.0066	0.039*	
C37	0.2171 (4)	0.57168 (18)	0.11825 (18)	0.0383 (8)	
H37	0.0988	0.5766	0.1023	0.046*	
C38	0.2706 (4)	0.54309 (18)	0.19819 (18)	0.0404 (8)	
H38	0.1886	0.5283	0.2375	0.048*	
C39	0.4426 (4)	0.53573 (18)	0.22183 (17)	0.0360 (7)	
H39	0.4778	0.5162	0.2772	0.043*	
C40	0.5622 (4)	0.55651 (16)	0.16571 (16)	0.0309 (7)	
H40	0.6803	0.5514	0.1820	0.037*	

### Atomic displacement parameters (Å^2^)

**Table:**

	*U*^11^	*U*^22^	*U*^33^	*U*^12^	*U*^13^	*U*^23^
Cl1	0.0833 (7)	0.0597 (6)	0.0763 (7)	−0.0261 (5)	−0.0084 (5)	0.0438 (5)
Cl2	0.0848 (7)	0.0409 (5)	0.0462 (5)	−0.0222 (5)	0.0028 (5)	−0.0050 (4)
Cl3	0.0769 (7)	0.0491 (5)	0.0696 (6)	−0.0043 (5)	−0.0137 (5)	−0.0286 (4)
Cl4	0.1054 (8)	0.0303 (4)	0.0463 (5)	−0.0209 (5)	−0.0075 (5)	0.0030 (4)
O1	0.0298 (12)	0.0424 (13)	0.0543 (13)	−0.0173 (10)	−0.0097 (10)	0.0186 (10)
O2	0.0322 (12)	0.0276 (10)	0.0331 (11)	−0.0145 (9)	0.0052 (9)	−0.0034 (8)
O3	0.0341 (13)	0.0340 (12)	0.0551 (14)	−0.0078 (10)	−0.0098 (11)	0.0013 (10)
O4	0.0251 (13)	0.0471 (13)	0.0630 (15)	−0.0032 (10)	−0.0036 (11)	−0.0115 (10)
O5	0.0375 (12)	0.0469 (12)	0.0257 (11)	−0.0194 (10)	0.0012 (9)	0.0010 (9)
O6	0.0306 (13)	0.0370 (12)	0.0395 (12)	−0.0076 (10)	−0.0017 (10)	0.0039 (9)
N1	0.0318 (15)	0.0284 (13)	0.0297 (14)	−0.0097 (11)	−0.0054 (12)	0.0054 (10)
N2	0.0255 (14)	0.0321 (14)	0.0408 (15)	−0.0094 (11)	−0.0037 (12)	0.0060 (11)
N3	0.0344 (15)	0.0337 (14)	0.0298 (14)	−0.0080 (12)	0.0021 (12)	0.0032 (11)
N4	0.0287 (15)	0.0385 (15)	0.0369 (15)	−0.0093 (12)	0.0032 (12)	0.0060 (12)
C1	0.038 (2)	0.0366 (19)	0.051 (2)	−0.0128 (15)	−0.0079 (16)	0.0214 (16)
C2	0.046 (2)	0.051 (2)	0.0362 (18)	−0.0143 (17)	−0.0057 (16)	0.0124 (16)
C3	0.0383 (19)	0.0326 (17)	0.0394 (19)	−0.0153 (14)	−0.0077 (15)	0.0063 (14)
C4	0.0253 (17)	0.0270 (16)	0.0347 (17)	−0.0109 (13)	−0.0077 (13)	0.0068 (13)
C5	0.0370 (19)	0.0335 (18)	0.0389 (18)	−0.0118 (15)	−0.0060 (15)	0.0087 (14)
C6	0.043 (2)	0.0284 (17)	0.058 (2)	−0.0119 (15)	−0.0089 (17)	0.0082 (15)
C7	0.0282 (18)	0.0258 (16)	0.0322 (17)	−0.0086 (14)	0.0007 (14)	−0.0001 (12)
C8	0.0270 (17)	0.0201 (14)	0.0273 (16)	−0.0073 (13)	−0.0053 (13)	0.0038 (12)
C9	0.0277 (17)	0.0190 (15)	0.0342 (17)	−0.0080 (13)	−0.0014 (15)	−0.0026 (12)
C10	0.0272 (18)	0.0259 (16)	0.0371 (18)	−0.0097 (13)	−0.0011 (14)	0.0048 (13)
C11	0.051 (2)	0.0453 (19)	0.0340 (18)	−0.0184 (16)	−0.0148 (15)	0.0109 (14)
C12	0.0244 (17)	0.057 (2)	0.0479 (19)	−0.0133 (15)	−0.0001 (15)	0.0107 (16)
C13	0.0290 (17)	0.0269 (16)	0.0332 (16)	−0.0149 (13)	0.0002 (13)	0.0015 (12)
C14	0.0321 (18)	0.0266 (16)	0.0232 (15)	−0.0088 (14)	0.0029 (14)	0.0015 (12)
C15	0.0316 (18)	0.0248 (16)	0.0251 (15)	−0.0096 (14)	0.0034 (13)	0.0007 (12)
C16	0.0399 (19)	0.0281 (16)	0.0288 (16)	−0.0121 (15)	0.0046 (14)	0.0039 (12)
C17	0.0413 (19)	0.0419 (19)	0.0301 (17)	−0.0155 (16)	0.0050 (14)	−0.0007 (14)
C18	0.054 (2)	0.044 (2)	0.0339 (18)	−0.0305 (18)	0.0074 (16)	−0.0073 (14)
C19	0.062 (2)	0.0218 (16)	0.0367 (18)	−0.0167 (16)	0.0106 (17)	−0.0006 (13)
C20	0.044 (2)	0.0312 (17)	0.0276 (16)	−0.0073 (15)	0.0020 (14)	−0.0006 (13)
C21	0.0355 (19)	0.0360 (19)	0.049 (2)	−0.0053 (15)	−0.0009 (16)	−0.0163 (15)
C22	0.0375 (19)	0.0366 (18)	0.0362 (18)	−0.0095 (15)	0.0034 (15)	−0.0054 (14)
C23	0.0354 (19)	0.0257 (16)	0.0411 (18)	−0.0097 (14)	0.0032 (15)	−0.0010 (14)
C24	0.0212 (16)	0.0280 (16)	0.0434 (19)	−0.0037 (13)	0.0034 (14)	−0.0044 (14)
C25	0.046 (2)	0.0309 (18)	0.051 (2)	−0.0005 (15)	−0.0095 (16)	0.0021 (15)
C26	0.051 (2)	0.0263 (17)	0.064 (2)	−0.0007 (16)	−0.0134 (18)	−0.0063 (17)
C27	0.0272 (18)	0.0234 (15)	0.0396 (18)	−0.0071 (14)	−0.0015 (15)	0.0041 (13)
C28	0.0236 (16)	0.0254 (15)	0.0282 (16)	−0.0048 (13)	0.0010 (13)	0.0022 (12)
C29	0.0286 (18)	0.0295 (16)	0.0269 (16)	−0.0083 (14)	−0.0027 (14)	0.0097 (13)
C30	0.0266 (17)	0.0325 (17)	0.0326 (17)	−0.0049 (14)	−0.0035 (14)	0.0097 (14)
C31	0.054 (2)	0.056 (2)	0.0412 (19)	−0.0060 (17)	0.0144 (17)	−0.0119 (16)
C32	0.0290 (18)	0.054 (2)	0.058 (2)	−0.0185 (16)	−0.0004 (16)	−0.0086 (16)
C33	0.0310 (17)	0.0367 (17)	0.0327 (17)	−0.0148 (14)	−0.0002 (14)	0.0033 (13)
C34	0.0332 (19)	0.0217 (15)	0.0300 (17)	−0.0084 (14)	−0.0062 (15)	−0.0013 (12)
C35	0.0259 (17)	0.0241 (15)	0.0278 (16)	−0.0059 (13)	−0.0019 (13)	0.0033 (12)
C36	0.0346 (19)	0.0360 (17)	0.0271 (16)	−0.0074 (14)	−0.0039 (15)	0.0024 (13)
C37	0.0258 (18)	0.0442 (19)	0.045 (2)	−0.0088 (15)	−0.0001 (15)	−0.0034 (15)
C38	0.042 (2)	0.0458 (19)	0.0353 (19)	−0.0152 (16)	0.0068 (16)	0.0015 (15)
C39	0.039 (2)	0.0395 (18)	0.0289 (17)	−0.0081 (15)	−0.0014 (15)	0.0032 (13)
C40	0.0329 (18)	0.0292 (16)	0.0311 (17)	−0.0074 (14)	−0.0035 (14)	−0.0009 (13)

### Geometric parameters (Å, º)

**Table:**

Cl1—C1	1.733 (3)	C15—C20	1.393 (3)
Cl2—C3	1.725 (3)	C16—C17	1.389 (4)
Cl3—C21	1.729 (3)	C16—H16	0.9500
Cl4—C23	1.734 (3)	C17—C18	1.379 (4)
O1—C7	1.221 (3)	C17—H17	0.9500
O2—C9	1.343 (3)	C18—C19	1.384 (4)
O2—C13	1.434 (3)	C18—H18	0.9500
O3—C14	1.221 (3)	C19—C20	1.381 (4)
O4—C27	1.232 (3)	C19—H19	0.9500
O5—C29	1.348 (3)	C20—H20	0.9500
O5—C33	1.424 (3)	C21—C22	1.368 (4)
O6—C34	1.220 (3)	C21—C26	1.377 (4)
N1—C9	1.339 (3)	C22—C23	1.371 (4)
N1—N2	1.368 (3)	C22—H22	0.9500
N1—C11	1.455 (3)	C23—C24	1.384 (4)
N2—C10	1.324 (3)	C24—C25	1.386 (4)
N3—C29	1.320 (3)	C24—C27	1.509 (4)
N3—N4	1.377 (3)	C25—C26	1.381 (4)
N3—C31	1.438 (3)	C25—H25	0.9500
N4—C30	1.323 (3)	C26—H26	0.9500
C1—C2	1.368 (4)	C27—C28	1.430 (4)
C1—C6	1.374 (4)	C28—C29	1.388 (3)
C2—C3	1.392 (4)	C28—C30	1.428 (4)
C2—H2	0.9500	C30—C32	1.490 (4)
C3—C4	1.376 (4)	C31—H31A	0.9800
C4—C5	1.388 (4)	C31—H31B	0.9800
C4—C7	1.503 (3)	C31—H31C	0.9800
C5—C6	1.377 (3)	C32—H32A	0.9800
C5—H5	0.9500	C32—H32B	0.9800
C6—H6	0.9500	C32—H32C	0.9800
C7—C8	1.453 (4)	C33—C34	1.516 (4)
C8—C9	1.398 (3)	C33—H33A	0.9900
C8—C10	1.425 (3)	C33—H33B	0.9900
C10—C12	1.488 (3)	C34—C35	1.476 (4)
C11—H11A	0.9800	C35—C36	1.385 (4)
C11—H11B	0.9800	C35—C40	1.394 (3)
C11—H11C	0.9800	C36—C37	1.379 (3)
C12—H12A	0.9800	C36—H36	0.9500
C12—H12B	0.9800	C37—C38	1.377 (4)
C12—H12C	0.9800	C37—H37	0.9500
C13—C14	1.507 (3)	C38—C39	1.383 (4)
C13—H13A	0.9900	C38—H38	0.9500
C13—H13B	0.9900	C39—C40	1.367 (3)
C14—C15	1.486 (4)	C39—H39	0.9500
C15—C16	1.390 (4)	C40—H40	0.9500
			
C9—O2—C13	119.17 (19)	C20—C19—H19	119.8
C29—O5—C33	120.3 (2)	C18—C19—H19	119.8
C9—N1—N2	112.0 (2)	C19—C20—C15	120.0 (3)
C9—N1—C11	128.4 (2)	C19—C20—H20	120.0
N2—N1—C11	119.4 (2)	C15—C20—H20	120.0
C10—N2—N1	104.7 (2)	C22—C21—C26	122.2 (3)
C29—N3—N4	112.6 (2)	C22—C21—Cl3	118.5 (2)
C29—N3—C31	126.8 (3)	C26—C21—Cl3	119.3 (2)
N4—N3—C31	120.4 (2)	C21—C22—C23	117.8 (3)
C30—N4—N3	104.7 (2)	C21—C22—H22	121.1
C2—C1—C6	122.0 (3)	C23—C22—H22	121.1
C2—C1—Cl1	118.5 (2)	C22—C23—C24	122.4 (3)
C6—C1—Cl1	119.4 (2)	C22—C23—Cl4	118.0 (2)
C1—C2—C3	118.3 (3)	C24—C23—Cl4	119.6 (2)
C1—C2—H2	120.9	C23—C24—C25	118.1 (3)
C3—C2—H2	120.9	C23—C24—C27	122.8 (2)
C4—C3—C2	121.4 (3)	C25—C24—C27	119.1 (3)
C4—C3—Cl2	119.8 (2)	C26—C25—C24	120.6 (3)
C2—C3—Cl2	118.8 (2)	C26—C25—H25	119.7
C3—C4—C5	118.3 (2)	C24—C25—H25	119.7
C3—C4—C7	122.7 (2)	C21—C26—C25	118.8 (3)
C5—C4—C7	118.8 (2)	C21—C26—H26	120.6
C6—C5—C4	121.3 (3)	C25—C26—H26	120.6
C6—C5—H5	119.4	O4—C27—C28	123.2 (3)
C4—C5—H5	119.4	O4—C27—C24	118.0 (2)
C1—C6—C5	118.6 (3)	C28—C27—C24	118.7 (3)
C1—C6—H6	120.7	C29—C28—C30	104.2 (2)
C5—C6—H6	120.7	C29—C28—C27	127.0 (3)
O1—C7—C8	124.4 (2)	C30—C28—C27	128.5 (3)
O1—C7—C4	117.5 (2)	N3—C29—O5	116.3 (2)
C8—C7—C4	117.9 (2)	N3—C29—C28	107.5 (2)
C9—C8—C10	102.8 (2)	O5—C29—C28	136.2 (3)
C9—C8—C7	127.3 (2)	N4—C30—C28	110.9 (3)
C10—C8—C7	129.5 (2)	N4—C30—C32	118.2 (3)
N1—C9—O2	118.3 (2)	C28—C30—C32	130.9 (3)
N1—C9—C8	108.0 (2)	N3—C31—H31A	109.5
O2—C9—C8	133.0 (3)	N3—C31—H31B	109.5
N2—C10—C8	112.4 (2)	H31A—C31—H31B	109.5
N2—C10—C12	118.5 (2)	N3—C31—H31C	109.5
C8—C10—C12	129.0 (3)	H31A—C31—H31C	109.5
N1—C11—H11A	109.5	H31B—C31—H31C	109.5
N1—C11—H11B	109.5	C30—C32—H32A	109.5
H11A—C11—H11B	109.5	C30—C32—H32B	109.5
N1—C11—H11C	109.5	H32A—C32—H32B	109.5
H11A—C11—H11C	109.5	C30—C32—H32C	109.5
H11B—C11—H11C	109.5	H32A—C32—H32C	109.5
C10—C12—H12A	109.5	H32B—C32—H32C	109.5
C10—C12—H12B	109.5	O5—C33—C34	112.6 (2)
H12A—C12—H12B	109.5	O5—C33—H33A	109.1
C10—C12—H12C	109.5	C34—C33—H33A	109.1
H12A—C12—H12C	109.5	O5—C33—H33B	109.1
H12B—C12—H12C	109.5	C34—C33—H33B	109.1
O2—C13—C14	111.3 (2)	H33A—C33—H33B	107.8
O2—C13—H13A	109.4	O6—C34—C35	122.4 (2)
C14—C13—H13A	109.4	O6—C34—C33	121.2 (2)
O2—C13—H13B	109.4	C35—C34—C33	116.5 (2)
C14—C13—H13B	109.4	C36—C35—C40	119.5 (2)
H13A—C13—H13B	108.0	C36—C35—C34	121.9 (2)
O3—C14—C15	122.1 (2)	C40—C35—C34	118.6 (2)
O3—C14—C13	120.0 (2)	C37—C36—C35	120.6 (3)
C15—C14—C13	117.8 (2)	C37—C36—H36	119.7
C16—C15—C20	119.2 (3)	C35—C36—H36	119.7
C16—C15—C14	121.8 (2)	C38—C37—C36	119.2 (3)
C20—C15—C14	119.0 (3)	C38—C37—H37	120.4
C17—C16—C15	120.6 (3)	C36—C37—H37	120.4
C17—C16—H16	119.7	C37—C38—C39	120.7 (3)
C15—C16—H16	119.7	C37—C38—H38	119.6
C18—C17—C16	119.7 (3)	C39—C38—H38	119.6
C18—C17—H17	120.2	C40—C39—C38	120.2 (3)
C16—C17—H17	120.2	C40—C39—H39	119.9
C17—C18—C19	120.1 (3)	C38—C39—H39	119.9
C17—C18—H18	120.0	C39—C40—C35	119.8 (3)
C19—C18—H18	120.0	C39—C40—H40	120.1
C20—C19—C18	120.5 (3)	C35—C40—H40	120.1
			
C9—N1—N2—C10	0.8 (3)	C16—C15—C20—C19	0.1 (4)
C11—N1—N2—C10	−174.7 (2)	C14—C15—C20—C19	179.9 (2)
C29—N3—N4—C30	1.7 (3)	C26—C21—C22—C23	−1.3 (4)
C31—N3—N4—C30	−173.2 (2)	Cl3—C21—C22—C23	178.0 (2)
C6—C1—C2—C3	2.3 (4)	C21—C22—C23—C24	0.2 (4)
Cl1—C1—C2—C3	−176.2 (2)	C21—C22—C23—Cl4	−178.7 (2)
C1—C2—C3—C4	−0.5 (4)	C22—C23—C24—C25	0.2 (4)
C1—C2—C3—Cl2	−179.1 (2)	Cl4—C23—C24—C25	179.1 (2)
C2—C3—C4—C5	−2.1 (4)	C22—C23—C24—C27	−177.7 (3)
Cl2—C3—C4—C5	176.5 (2)	Cl4—C23—C24—C27	1.2 (4)
C2—C3—C4—C7	174.2 (3)	C23—C24—C25—C26	0.5 (4)
Cl2—C3—C4—C7	−7.2 (4)	C27—C24—C25—C26	178.4 (3)
C3—C4—C5—C6	2.9 (4)	C22—C21—C26—C25	1.9 (5)
C7—C4—C5—C6	−173.5 (3)	Cl3—C21—C26—C25	−177.4 (2)
C2—C1—C6—C5	−1.5 (5)	C24—C25—C26—C21	−1.5 (5)
Cl1—C1—C6—C5	177.0 (2)	C23—C24—C27—O4	108.3 (3)
C4—C5—C6—C1	−1.2 (4)	C25—C24—C27—O4	−69.5 (3)
C3—C4—C7—O1	−74.3 (4)	C23—C24—C27—C28	−74.4 (3)
C5—C4—C7—O1	102.0 (3)	C25—C24—C27—C28	107.8 (3)
C3—C4—C7—C8	109.1 (3)	O4—C27—C28—C29	−16.2 (4)
C5—C4—C7—C8	−74.6 (3)	C24—C27—C28—C29	166.7 (2)
O1—C7—C8—C9	−20.3 (4)	O4—C27—C28—C30	156.0 (3)
C4—C7—C8—C9	156.0 (2)	C24—C27—C28—C30	−21.1 (4)
O1—C7—C8—C10	167.9 (3)	N4—N3—C29—O5	178.15 (19)
C4—C7—C8—C10	−15.8 (4)	C31—N3—C29—O5	−7.4 (4)
N2—N1—C9—O2	−173.02 (19)	N4—N3—C29—C28	−0.6 (3)
C11—N1—C9—O2	2.0 (4)	C31—N3—C29—C28	173.9 (2)
N2—N1—C9—C8	−1.2 (3)	C33—O5—C29—N3	145.0 (2)
C11—N1—C9—C8	173.8 (2)	C33—O5—C29—C28	−36.8 (4)
C13—O2—C9—N1	−127.1 (2)	C30—C28—C29—N3	−0.7 (3)
C13—O2—C9—C8	63.6 (3)	C27—C28—C29—N3	173.0 (2)
C10—C8—C9—N1	1.1 (3)	C30—C28—C29—O5	−179.0 (3)
C7—C8—C9—N1	−172.5 (2)	C27—C28—C29—O5	−5.3 (5)
C10—C8—C9—O2	171.2 (3)	N3—N4—C30—C28	−2.1 (3)
C7—C8—C9—O2	−2.4 (4)	N3—N4—C30—C32	179.1 (2)
N1—N2—C10—C8	−0.1 (3)	C29—C28—C30—N4	1.8 (3)
N1—N2—C10—C12	−177.7 (2)	C27—C28—C30—N4	−171.8 (2)
C9—C8—C10—N2	−0.6 (3)	C29—C28—C30—C32	−179.6 (3)
C7—C8—C10—N2	172.8 (3)	C27—C28—C30—C32	6.8 (4)
C9—C8—C10—C12	176.7 (3)	C29—O5—C33—C34	−52.9 (3)
C7—C8—C10—C12	−9.9 (4)	O5—C33—C34—O6	−2.0 (3)
C9—O2—C13—C14	76.7 (3)	O5—C33—C34—C35	177.3 (2)
O2—C13—C14—O3	−14.8 (3)	O6—C34—C35—C36	−165.8 (2)
O2—C13—C14—C15	166.1 (2)	C33—C34—C35—C36	14.8 (4)
O3—C14—C15—C16	−176.7 (3)	O6—C34—C35—C40	13.6 (4)
C13—C14—C15—C16	2.4 (4)	C33—C34—C35—C40	−165.7 (2)
O3—C14—C15—C20	3.5 (4)	C40—C35—C36—C37	−0.8 (4)
C13—C14—C15—C20	−177.4 (2)	C34—C35—C36—C37	178.7 (2)
C20—C15—C16—C17	−0.5 (4)	C35—C36—C37—C38	0.5 (4)
C14—C15—C16—C17	179.7 (2)	C36—C37—C38—C39	0.0 (4)
C15—C16—C17—C18	0.1 (4)	C37—C38—C39—C40	−0.3 (4)
C16—C17—C18—C19	0.8 (4)	C38—C39—C40—C35	0.0 (4)
C17—C18—C19—C20	−1.2 (4)	C36—C35—C40—C39	0.5 (4)
C18—C19—C20—C15	0.7 (4)	C34—C35—C40—C39	−179.0 (2)

### Hydrogen-bond geometry (Å, º)

Cg3 and Cg6 are the centroids of the C15–C20 and C35–C40 rings, respectively.

**Table:**

*D*—H···*A*	*D*—H	H···*A*	*D*···*A*	*D*—H···*A*
C12—H12*A*···O1^i^	0.98	2.42	3.353 (4)	159
C32—H32*A*···O4^i^	0.98	2.45	3.420 (4)	168
C16—H16···N2^ii^	0.95	2.51	3.414 (4)	159
C36—H36···N4^ii^	0.95	2.54	3.334 (4)	141
C37—H37···O6^ii^	0.95	2.55	3.490 (4)	172
C25—H25···O4^iii^	0.95	2.56	3.302 (4)	135
C39—H39···O3^iv^	0.95	2.54	3.346 (4)	143
C33—H33*A*···*Cg*6^v^	0.99	2.97	3.683 (3)	130
C38—H38···*Cg*3^vi^	0.95	2.68	3.499 (3)	145

Symmetry codes: (i) *x*+1, *y*, *z*; (ii) *x*−1, *y*, *z*; (iii) −*x*+1, −*y*+2, −*z*; (iv) −*x*+1, −*y*+1, −*z*+1; (v) −*x*+1, −*y*+1, −*z*; (vi) −*x*, −*y*+1, −*z*+1.
